# CSC-3436 switched tamoxifen-induced autophagy to apoptosis through the inhibition of AMPK/mTOR pathway

**DOI:** 10.1186/s12929-016-0275-y

**Published:** 2016-08-15

**Authors:** Sheng-Tang Wu, Guang-Huan Sun, Tai-Lung Cha, Chien-Chang Kao, Sun-Yran Chang, Sheng-Chu Kuo, Tzong-Der Way

**Affiliations:** 1 Division of Urology, Department of Surgery, Tri-Service General Hospital and National Defense Medical Center, Taichung, Taiwan; 2 School of Pharmacy, College of Pharmacy, China Medical University, Taichung, Taiwan; 3 Department of Biological Science and Technology, College of Biopharmaceutical and Food Sciences, China Medical University, No. 91 Hsueh-Shih Road, Taichung, 40402 Taiwan R.O.C; 4 Department of Health and Nutrition Biotechnology, College of Health Science, Asia University, Taichung, Taiwan; 5 Graduate institute of Pharmaceutical Chemistry, China Medical University, Taichung, 40402 Taiwan R.O.C

**Keywords:** Triple-negative breast cancer, Tamoxifen, CSC-3436, Endoplasmic reticulum stress, AMPK/mTOR

## Abstract

**Background:**

Triple-negative breast cancer (TNBC) lacks specific therapeutic target and limits to chemotherapy and is essential to develop novel therapeutic regimens. Increasing studies indicated that tamoxifen, a selective estrogen receptor modulators (SERMs), has anti-tumor therapeutic effect in estrogen receptor α (ERα)-negative tumor. Here, we determined whether autophagy was activated by tamoxifen in TNBC cells. Moreover, CSC-3436 displayed strong and selective growth inhibition on cancer cells. Next, we investigated the anti-proliferation effect of combination of CSC-3436 plus tamoxifen on cell death in TNBC cells.

**Results:**

Our study found that tamoxifen induces autophagy in TNBC cells. Endoplasmic reticulum stress and AMPK/mTOR contributed tamoxifen-induced autophagy. Interestingly, in combination treatment with CSC-3436 enhanced the anti-proliferative effect of tamoxifen. We found that CSC-3436 switched tamoxifen-induced autophagy to apoptosis via cleavage of ATG-5. Moreover, AMPK/mTOR pathway may involve in CSC-3436 switched tamoxifen-induced autophagy to apoptosis. The combination of tamoxifen and CSC-3436 produced stronger tumor growth inhibition compared with CSC-3436 or tamoxifen alone treatments in vivo.

**Conclusion:**

These data indicated that CSC-3436 combined with tamoxifen may be a potential approach for treatment TNBC.

## Background

Hormone receptors (estrogen and progesterone receptors) and human epidermal growth factor receptor (HER2) status are biological markers and are widely accepted in terms of their clinical importance in breast cancer. Triple-negative breast cancer (TNBC) is defined by a lack expression of all three receptors (estrogen, progesterone and HER2) and represents approximately 15–20 % of all breast cancers [1–3]. TNBC patients are high risk of early recurrence and high incidence of visceral and central nervous system (CNS) metastases [4]. TNBC lacks specific therapeutic target and limits to chemotherapy. However, the data about TNBC therapy issue are insufficient as present. It is essential to develop novel therapeutic regimens.

Tamoxifen has represented the widely used therapy with its abundant, mature safety data in clinical hormone therapy [5]. Tamoxifen belongs to selective estrogen receptor modulators (SERMs) and acts as antagonist that complete with estradiol binding to estrogen receptor α (ERα) and then could modulate its transcriptional capabilities for ERα-positive breast cancer patients [6, 7]. There have been increasing studies indicated that tamoxifen has anti-tumor therapeutic effect in both ERα-positive breast cancer and ERα-negative tumors e.g. glioma, melanoma, colon cancer and pancreatic carcinoma though growth inhibition, apoptosis and autophagy, suggesting that tamoxifen could be an anther choice for ERα-negative tumor treatment [8].

In principle, autophagy is a highly-conserved catabolic in eukaryotic cells and it maintains cellular homeostasis through the balance of cellular metabolism and the clearance of aggregated or misfolded proteins [9]. Autophagy-deregulated related to many diseases, such as neurodegeneration, heart disease and cancer development. Briefly, the process of autophagy is featured by the formation of double-membrane cytosolic vesicles, known as autophagosomes. The autophagosomes fuse with lysosomes to form autolysosome, in which lysosomal hydrolases digest the cargo to metabolites that are released back into the cytosol for recycling [10]. It well known that the effect of the formation of autophagosome include autophagy-related gene (ATG) 5, Beclin-1 (known as ATG6) and the conversion of LC3-I to LC3-II through proteolytic cleavage and lipidaation is considerate a hallmark of mammalian autophagy [11–13]. Autophagy plays a dual role in cancer process dependents on various cases. Autophagy plays pro-death response by autophagic cell death. On the contrary, autophagy exhibits cytoprotective response undendoplasmic reticulum stress conditions involving nutritional starvation of amino acids or fatty acids, hypoxia, oxidative stress, damaged mitochondria and chemotherapies [11, 14].

Previous studies indicated that cytoprotective autophagy provided cancer cell chemoresistance and that autophagy suppression enhanced therapy-induced apoptosis and proliferation inhibition [15–19]. The relationship between autophagy and apoptosis is unclear. A rationale for the use of autophagy inhibitors in combination with chemotherapy agents is considered a better approach to improve efficacy of anticancer therapeutics. Therefore, investigating the autophagy inhibition enhance cancer cell chemosensitivity is considered a better approach to improve efficacy of anticancer therapeutics during the tumor development.

In our previous studies, CSC-3436 displays strong and selective growth inhibition on the NCI60 cell lines by inhibiting tubulin polymerization [20]. The IC_50_ of CSC-3436 in different types of breast cancer cell lines including TNBC (MDA-MB-231, MDA-MB-468, BT-549, and BT-20), ERα-positive (MCF-7 and T47D) and HER-2 amplified (BT-474) narrow down to nanomolar. In the present study, we found that tamoxifen induced cytoptotective role in TNBC cells. Interestingly, CSC-3436 sensitized TNBC cells to tamoxifen.

## Methods

### Cell culture

TNBC cell, MDA-MB-231, is mesenchymal phenotype with high vimentin expression but undetectable E-cadherin expression. MDA-MB-231 was grown in DMEM/F12 (Invitrogen Corporation, Carlsbad, CA, USA). Medium was supplemented with 10 % fetal bovine serum (FBS), 2 mM L-glutamine, 100 U penicillin and 100 μg streptomycin (Invitrogen Corporation, Carlsbad, CA, USA). All cell lines were grown in a humidified incubator at 37 °C under 5 % CO_2_ in air.

### Reagents and antibodies

CSC-3436 was resuspended in DMSO. 3-(4,5-dimethylthiazol-2-yl)-2,5-diphenyl tetrazolium bromide (MTT), 3-methyladenine [3-MA (autophagy inhibitor)] and primary antibody β-actin were purchased from Sigma Chemical Co. (St. Louis, Mo, USA). Salubrinal (endoplasmic reticulum stress inhibitor), ISP-1 (ceramide inhibitor), primary antibodies cleavage ATG-5 and primary GRP78 were purchased from Santa Cruz Biotechnology (Santa Cruz, CA, USA). Primary antibodies Beclin-1, LC3-I/II, ATG5-ATG12 complex, p-AMPK, AMPK, p-mTOR, mTOR and β-actin were purchased from Cell Signaling Technology (Beverly, MA, USA). Secondary antibodies, HRP-conjugated Goat anti-Mouse IgG and Goat anti-Rabbit IgG, were obtained from Millipore (Billerica, MA, USA).

### Supravital cell staining with acridine orange (AO) detection

To detect acidic vesicular organelles (AVOs), cells were stained with 1 mg/mL AO (Sigmal Chemical Co.,) for a period 20 min. Cells were then analyzed with a fluorescent microscope (Nikon TE2000-U) using an excitation filter of 502 nm and an emission filter of 525 nm.

### Monodansylcadaverine staining

After various treatments for autophagy induction, cells were stained with 5 mM monodansylcadaverine (MDC; Fluka) for a period 15 min. Cells were than analyzed a fluorescent microscope (Nikon TE2000-U) using an excitation filter of 360 nm and an emission filter of 525 nm.

### Cell viability assay

MDA-MB-231 cells were seeded in a 24-well plate (2 × 10^4^ cells/well) overnight, and then were treated with indicated times of CSC-3436 with or without tamoxifen or tamoxifen only. Cell viability was examined by the MTT assay. Briefly, 80 μL MTT solution (2 mg/mL) was added to each well to make a final volume of 500 μL and incubated for 1.5 h at 37 °C. The supernatant was aspirated, and the MTT-formazan crystals formed by metabolically viable cells were dissolved in 500 μL of DMSO. Finally, the absorbance at O.D. 570 nm was detected by enzyme-linked immunosorbent assay (ELISA) reader.

### Western blot analysis

Cells were seeded onto a 100-mm culture dishes (1 × 10^6^/dish) containing 10 % FBS. Cells were than treated with various agents as indicated in the figure captions. After treatment, the total proteins were extracted by adding 50 μL of gold lysis buffer (50 mM Tris–HCl, pH 7.4; 1 mM NaF; 150 mM NaCl; 1 mM EGTA; 1 mM phenylmethylsulfonyl floride; 1 % NP-40; and 10 mg/mL leupeptin) to the cell pellets. Lysate proteins were determined by the Lowry protein assay (Bio-Rad Laboratories). The samples (50 μg of proteins) of total cell lysates were resolved by sodium dodecyl sulfate-polyacrylamide gel electrophoresis and (SDS-PAGE), transferred to nitrocellulose membranes. Membranes were blocked with 5 % BSA (Sigma, St. Louis, MO, USA) for 1 h at room temperature, and probed with primary antibody for 1.5 h at room temperature or overnight at 4 °C followed by HRP-conjugated appropriated secondary antibodies.

### Cell cycle analysis

MDA-MB-231 cells were suspended with ice-cold PBS and fixed in 75 % ethanol at −20 °C for 18 h. After fixation, the cells were washed twice, incubated in 0.5 mL of 0.5 % Triton X-100/PBS at 37 °C for 30 min with 1 mg/mL of RNase A, and stained with 0.5 mL of 50 μg/mL propidium iodide (PI) for 10 min. Fluorescence emitted from the PI-DNA complex was analyzed at 488 nm/600 nm (excitation/emission wavelength) by a fluorescence activated cell sorter (FACScan flow cytometry). The population of nuclei in each phase of the cell cycle was determined using Cell Quest and analyzed by WinMDI software programs (BectonDickinson, San Jose, California).

### RNA interference

We used the MicroPorator, a pipette-type electroporation system, (Digital Bio Tech, Korea) to transfect cells. The RNA interference reagents were obtained from the National RNA interference Core Facility located at the Institute of Molecular Biology/Genomic Research Centre, Academia Sinica. The human library is referred to as TRC-Hs 1.0. ATG5 clone is identified as short hairpin RNA (shRNA) TRCN0000330394.

### Animal studies

Female BALB/c nude mice (18–20 g; 6–8 weeks of age) were purchased from the National Animal Center (Taipei, Taiwan) and maintained in pressurized ventilated cage in accordance with institutional regulations. Animal studies involving mice were approved by the Institutional Animal Care and Use Committee (IACUC) of China Medical University and Reg. No. 101-15-N. All efforts were made to minimize animal suffering and to reduce the number of animals sacrificed.

MDA-MB-231 cells (3 × 10^6^) were inoculated subcutaneous into the right flank of the mice. Seven days after inoculation, when tumor volumes were larger than 100 mm^3^, the mice were divided into indicated groups (nine mice per group) and treated daily with vehicle alone and various doses of indicated drugs. The mice were weighed, and their tumors were measured using calipers twice per week by using a digital caliper. The tumor volume was calculated using the following formula: (width × length^2^)/2. On the final day of treatment, the mice were sacrificed; the tumors were excised, weighed, and sectioned; the tumor sections were embedded in an optimal cutting temperature (OCT) compound and frozen at −70 °C.

### Statistical analysis

One-way analysis of variance (ANOVA) was used for the comparison of more than two mean values. Results represent at least two to three independent experiments. Results with a *P* value less than 0.05 were considered statistically significant. *, *p* < 0.05.

## Results

### Tamoxifen induces autophagy in TNBC cells

**Fig. 1:**
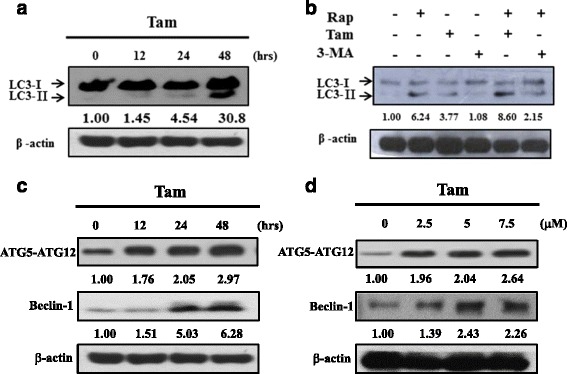
Tamoxifen induces autophagy in MDA-MB-231 cells. **a**, **c** MDA-MB-231 cells were incubated with tamoxifen (5 μM) for 12, 24 and 48 h. Cells were then harvested and lysed for the detection of LC3I/II, Beclin-1, ATG5-ATG12 complex and β-actin. **b** MDA-MB-231 cells were incubated with rapamycin, tamoxifen, and 3-MA for 24 h. Cells were then harvested and lysed for the detection of LC3-I/II and β-actin. **d** MDA-MB-231 cells were incubated with various concentrations (0, 2.5, 5 and 7.5 μM) for 48 h. Beclin-1, ATG5-ATG12 complex and β-actin were determined by western blotting

Tamoxifen is a well-known agent to trigger ERα-positive MCF-7 breast cells autophagy [21]. To determine whether autophagy is activated by tamoxifen in ERα-negative breast cells, we harvested TNBC MDA-M231 cells at the indicated times after exposure to 5 μM tamoxifen. LC3 is essential for autophagosome formation and as an autophagy marker [11]. Western blot analysis of LC3-I and LC3-II was determined by western blotting. Tamoxifen triggered the conversion of LC3 from LC3-I to LC3-II in MDA-MB-231 cells (Fig. 1a). MDA-MB-231 cells treated with tamoxifen, rapamycin (autophagy inducer), and 3-MA (autophagy inhibitor) to confirm tamoxifen-induced LC3-II accumulation. 3-MA suppressed partly tamoxifen-induced LC3-II accumulation (Fig. 1b). To investigate whether two autophagy-related proteins, Beclin-1 and ATG5-ATG12 complex, were involved in tamoxifen-induced autophagy. MDA-MB-231 cells were treated with tamoxifen at various concentrations (0, 2.5, 5 and 7.5 μM) for 48 h, or at the indicated times (0, 12, 24 and 48 h) after exposure to 5 μM tamoxifen. Tamoxifen treated increased Beclin-1 and ATG5-ATG12 complex protein levels both in dose- and time-dependent manner (Fig. 1c and d).

### Tamoxifen-induced AV formation and MDC-stained autophagosomes/autophagic bodies

**Fig. 2:**
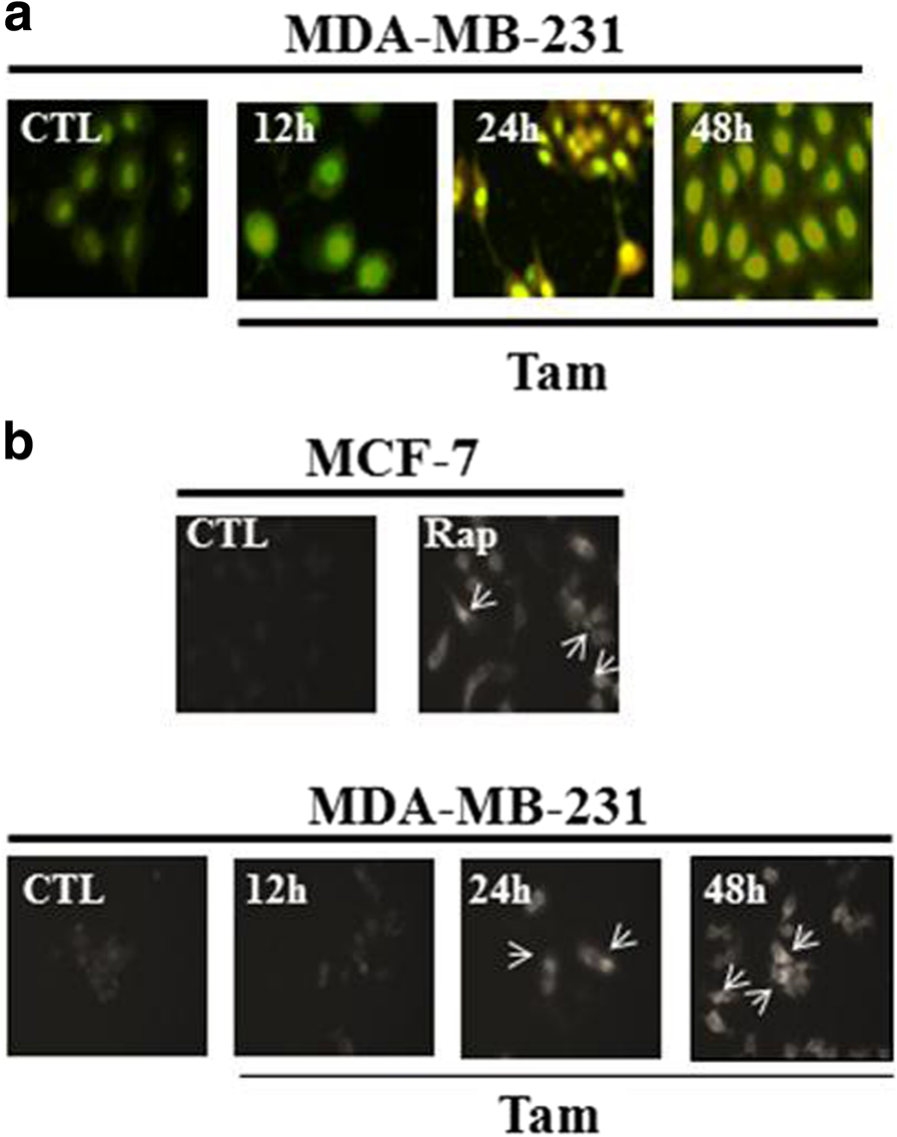
AO and MDC for autophagy detection in MDA-MB-231 cells. **a** Immunofluorescence microscopy of acridine orange (AO)-stained MDA-MB-231 cells treated with tamoxifen for 0, 12, 24 and 48 h. **b** Monodansylcadaverine (MDC)-labeled vesicles (indicated by arrows) were incubated by rapamycin in MCF-7 (as a positive control), 5 μM tamoxifen for 0, 12, 24 and 48 h in MDA-MM-231 cells

To observe autophagic vacuoles (AV) formation, MDA-MB-231 cells were treated with indicated times (0, 12, 24 and 48 h) of tamoxifen and analyzed cells by using confocal microscopy. The number of LC3(+) vacuoles continued to increase for 12 to 48 h after initiation of tamoxifen treatment (Fig. 2a). MDC is an autofluorescent agent and as a specific autophagolysosome marker [22] to analyze the autophagic process. MDA-MB-231 cells were treated with 5 μM tamoxifen at 0, 12, 24 and 48 h and analyzed cells by using microscopy. Arrows indicated MDC-stained autophagosomes/autophagic bodies. The AV continued to increase for 24 to 48 h after initiation of tamoxifen treatment (Fig. 2b). Taken together, tamoxifen induces ERα-negative breast cancer cells autophagy.

### Tamoxifen-induced autophagy related with endoplasmic reticulum stress and AMPK/mTOR pathway

**Fig. 3:**
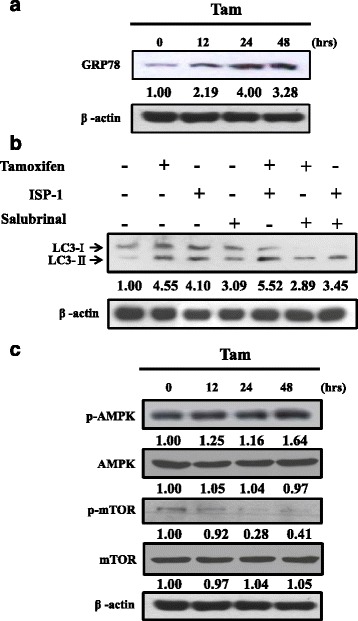
Tamoxifen-induced autophagy is mediated through endoplasmic reticulum stress and AMPK/mTOR signaling. MDA-MB-231 cells were incubated with 5 μM tamoxifen for 12, 24 and 48 h. Cells were then harvested and lysed for the detection of **a** GRP78 and **c** p-AMPK, AMPK, p-mTOR, mTOR and β-actin. **b** MDA-MB-231 cells were in the presence of tamoxifen (5 μM), salubrnal (5 μM) and/or ISP-1(10 μM) for 48 h followed by analysis of LC3 expression by western blotting

Previous studies indicated that endoplasmic reticulum stress and ceramide contributed to autophagy induction [23, 24]. Therefore, we determined whether endoplasmic reticulum stress and ceramide generation participated in autophagy induction by tamoxifen in MDA-MB-231 cells. MDA-MB-231 cells were treated with several times (0, 12, 24 and 48 h) after exposure to 5 μM tamoxifen. GRP78, the endoplasmic reticulum stress marker, was up-regulation (Fig. 3a). We next determined the effect of the endoplasmic reticulum stress inhibitor salubrinal on tamoxifen-induced autophagy by analyzing LC3-II accumulation. Salubrinal inhibited tamoxifen-induced LC3-II accumulation. We also examined the effect of the ceramide inhibitor ISP-1 on tamoxifen-induced autophagy but LC3-II accumulation didn’t significantly change (Fig. 3b). Autophagy is promoted by AMPK, which is a key energy sensor to regulate energy homeostasis and also inhibits the mammalian target of rapamycin (mTOR) [25]. MDA-MB-231 cells were treated with several times (0, 12, 24 and 48 h) after exposure to 5 μM tamoxifen and p-AMPK was up-regulation and p-mTOR was down-regulation (Fig. 3c). Taken together, endoplasmic reticulum stress and AMPK/mTOR contributed tamoxifen-induced autophagy.

### CSC-3436 plus tamoxifen enhanced cell death in MDA-MB-231 cells

**Fig. 4:**
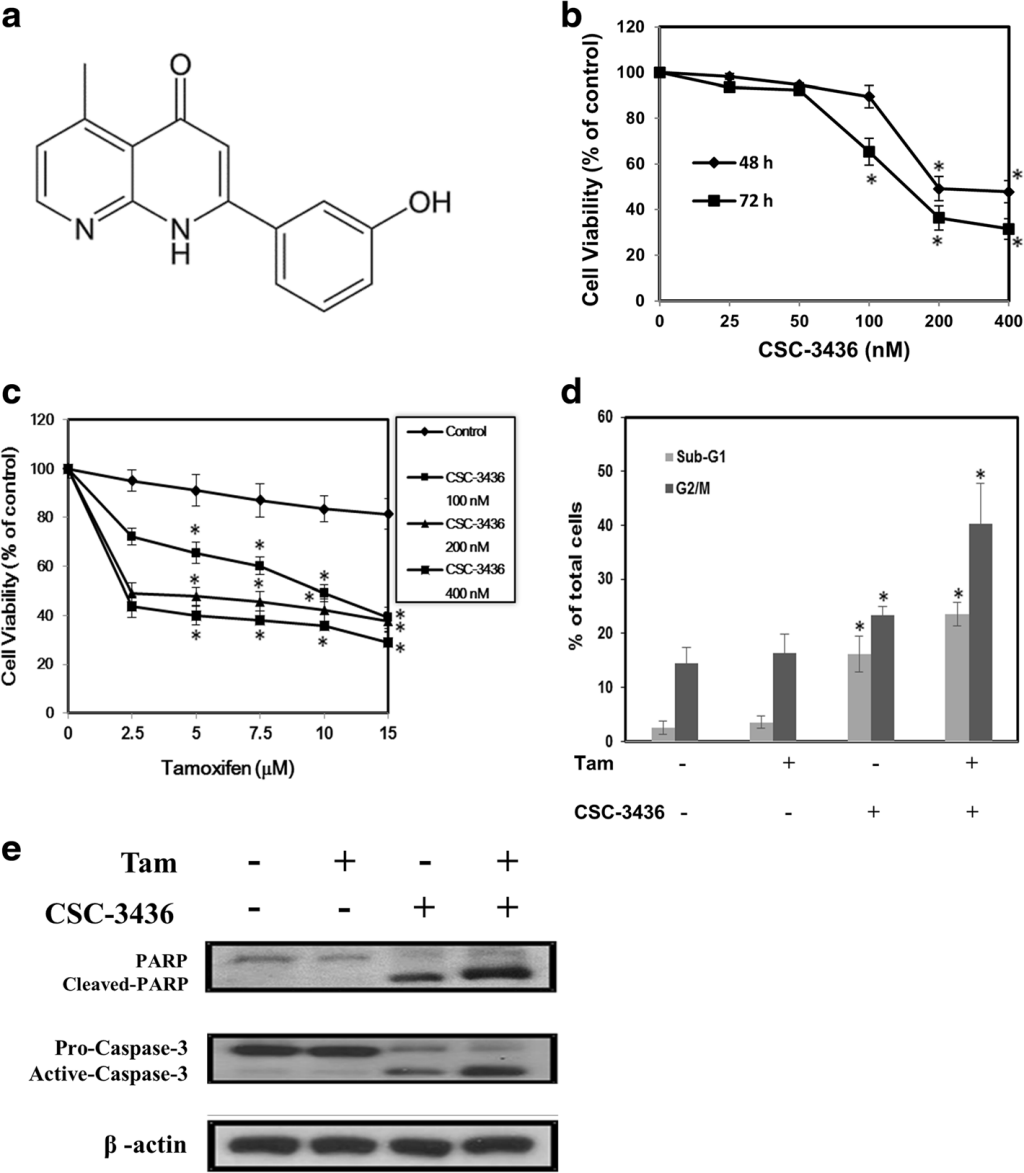
Combinatorial treatment with CSC-3436 and tamoxifen induced stronger G2/M phase arrest and anti-proliferative ability. **a** Chemical structure of CSC-3436. **b** MDA-MB-231 cells were treated with several concentrations (0, 25, 50, 100, 200 and 400 nM) CSC-3436 for 48 and 72 h. Cell proliferation was assessed using the MTT assay. The percentage of cell growth inhibition was calculated by the absorption of control cells as 100 %. **c** MDA-MB-231 cells were treated with various concentrations (0, 2.5, 5, 7.5, 10 and 15 μM) tamoxifen alone or in the presence of CSC-3436 (100, 200, and 400 nM) for 48 h. Cell proliferation was assessed using the MTT assay. The percentage of cell growth inhibition was calculated by the absorption of control cells as 100 %. **d** MDA-MB-231 cells were treated with CSC-3436 (200 nM) and tamoxifen (2.5 μM) alone or CSC-3436 (200 nM) plus tamoxifen (2.5 μM) for 48 h. Sub-G1 and G2/M phase were assay by PI staining. *p < 0.05 compared with control group. **e** MDA-MB-231 cells were treated with CSC-3436 (200 nM) and tamoxifen (2.5 μM) alone or CSC-3436 (200 nM) plus tamoxifen (2.5 μM) for 48 h followed by analysis of PARP and Caspase-3 by western blotting

Firstly, we examined the effect of CSC-3436 on cell growth inhibition in MDA-MB-231 cells. MDA-MB-231 cells were treated with CSC-3436 (Fig. 4a) at various concentrations (25, 50, 100, 200 and 400 nM) for 48 and 72 h. CSC-3436 induced growth inhibition both in dose- and time-dependent manner (IC_50_ = 205 ± 3.21 for 48 h, IC_50_ = 148 ± 2.31 for 72 h, respectively) (Fig. 4b). Next, we investigated the anti-proliferation effect of CSC-3436, tamoxifen, or combination of CSC-3436 plus tamoxifen on cell death in MDA-MB-231 cells. The combination treatment with CSC-3436 enhanced the anti-proliferative effect of tamoxifen (Fig. 4c). The PI staining indicated that the proportion of cells in apoptosis in MDA-MB-231 cells treated with CSC-3436 plus tamoxifen was higher as compared with tamoxifen treated alone (Fig. 4d). To test whether CSC-3436 plus tamoxifen enhanced apoptosis in MDA-MB-231 cells, we conducted Western blotting. Our result indicated that the cleavage patterns of PARP and caspase-3 were observed in the combination of CSC-3436 plus tamoxifen (Fig. 4e). These data indicated that CSC-3436 switched tamoxifen-induced autophagy to apoptosis.

### ATG-5 and AMPK/mTOR pathway involved in CSC-3436 switched tamoxifen- induced autophagy to apoptosis

**Fig. 5:**
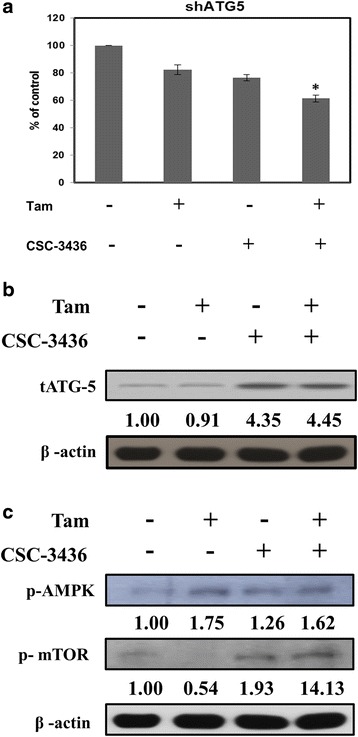
Relationship between autophagy and apoptosis induced by CSC-3436 and tamoxifen. **a** MDA-MB-231 cells were transfected with the shATG5 and then treated with CSC-3436 (200 nM) and tamoxifen (2.5 μM) alone or CSC-3436 (200 nM) plus tamoxifen (2.5 μM) for 24 h. Cell proliferation was assessed using the MTT assay. The percentage of cell growth inhibition was calculated by the absorption of control cells as 100 %. *p < 0.05 compared with control group. MDA-MB-231 cells were treated with CSC-3436 (200 nM) and tamoxifen (2.5 μM) alone or CSC-3436 (200 nM) plus tamoxifen (2.5 μM) for 48 h. The cleavage of **b** tATG5 and **c** p-AMPK and p-mTOR and β-actin were determined by western blotting

Autophagy under various stressful conditions could play a pro-survival or pro-death role [11]. ATG-5 is required for the generation of autophagosome in autophagy process [26]. To clarify the role of ATG-5 in tamoxifen-induced autophagy, we knocked down ATG-5 expression with shATG5 in MDA-MB-231 cell and examined the cell viability by MTT assay. ATG-5-deficient MDA-MB-231 cells exhibited that CSC-3436 plus tamoxifen treated reduced higher cell viability than tamoxifen or CSC-3436 treated alone (Fig. 5a). Previous report indicates that cleavage of ATG-5 involved in autophagy switch to apoptosis process [23, 27]. We investigated whether CSC-3436 switched tamoxifen-induced autophagy to apoptosis via cleavage of ATG-5. We performed western blotting assay to measure the cleavage of ATG-5. CSC-3436 alone or CSC-3436 plus tamoxifen exhibited the ATG-5 cleavage product (Fig. 5b). We also examined the effect of CSC-3436 on tamoxifen-regulated p-AMPK and p-mTOR. CSC-3436 inhibited tamoxifen-induced up-regulation of p-AMPK slightly and down-regulation of p-mTOR protein level expressions (Fig. 5c). Taken together, the cleavage of ATG-5 and AMPK/mTOR pathway may involve in CSC-3436 switched tamoxifen-induced autophagy to apoptosis.

### In vivo efficacy of the combination of tamoxifen and CSC-3436 in MDA-MB-231 tumor xenograft model

**Fig. 6:**
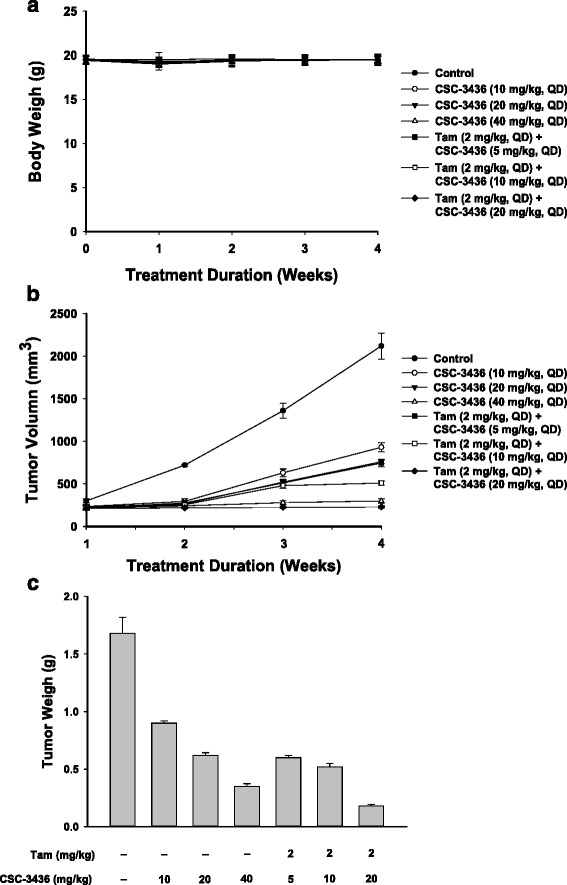
Effect of the combinational treatment with tamoxifen and CSC-3436 on tumor growth in MDA-MB-231 xenograft model. Each 5 week-old nude mouse was performed by s. c. injection of MDA-MB-231 cells. Mice were randomly assigned to seven groups (*n* = 9), and oral administrations treated with tamoxifen (2 mg/kg), CSC-3436 (10, 20 and 40 mg/kg, respectively) and the tamoxifen (2 mg/kg) plus CSC-3436 (5, 10, and 20 mg/kg, respectively). **a** Mean body weights, **b** mean tumor volumes, **c** tumor weights for each treatment group. *p < 0.05 compared with vehicle-treated group

To further check the in vivo relevance of the above observations, we examined the antitumor activity of the combinatorial treatment with CSC-3436 and tamoxifen in nude mice bearing MDA-MB-231 tumor xenografts (starting mean tumor volume, 100 ± 5 mm^3^). The tumor bearing mice were either treated oral administration with vehicle, CSC-3436 (10, 20 and 40 mg/kg) or CSC-3436 (5, 10 and 20 mg/kg) combined with tamoxifen (2 mg/kg). There was no significant change in body weights compared with the vehicle-treated group in all treatments (Fig. 6a). Previous study indicated that tamoxifen treatment alone was disappointed to anti-tumor activity of MDA-MB-231 tumor xenograft model [28]. Treatment with CSC-3436 alone induced a dose dependent inhibition in comparison to the vehicle-treated group. Tamoxifen combined with CSC-3436 at low (5 mg/kg), medium (10 mg/kg) and high (20 mg/kg) doses exhibited much more significant tumor growth inhibition (Fig. 6b). Apparently, CSC-3436 (5 mg/kg) plus tamoxifen-group reached similar tumor volume inhibition to CSC-3436 (20 mg/kg)-treated group. The tumor weights had the similar results in tumor volume inhibition (Fig. 6c). Thus, combination treatment of tamoxifen and CSC-3436 produced stronger tumor growth inhibition in comparison to the CSC-3436 or tamoxifen alone treatments.

## Discussion

Current data suggest that autophagy targeted provide therapeutic benefits to patients. Failure to apoptosis induction in cancer cells is one of main factor to cause chemotherapy resistance. As cells fail to undergo apoptosis, some anti-cancer agents such as etoposide could induce cell death by autophagy. On the other words, failure to induce apoptosis may drive cells undergo another cell death mechanism, autophagy [29–31]. Besides, a number of studies have been demonstrated that autophagy suppression enhances a result of chemotherapy by increasing apoptosis [32–36]. However, the relationship between autophagy and apoptosis in cell death on cancer cells remains poorly understood.

Tamoxifen is one of highly successful adjuvant endocrine drug for over 30 year due to its abundant, mature safety data in clinical [5] and function as an antagonist via competition with estradiol to bind to ERα and modulation of gene expression for ERα-positive breast cancer cells [37, 38]. Although tamoxifen used for ERα-positive breast cancer patients, it also shows therapeutic activity in ERα-negative cancer [39, 40]. Other studies suggested that a high dose of tamoxfein (approximately four fold to eight fold higher than that for ERα-positive breast cancers) exhibits anti-tumor activity in ERα-negative cancer cells such as glioma, melanoma and pancreatic carcinoma [41–43]. Besides to induced growth inhibition and apoptosis, there have been increasing reports showing that tamoxifen induced autophagy in breast and colon cancer [8, 24]. Moreover, tamoxifen induced both ERα-positive MCF-7 and ERα-negative SKBr-3 breast cancer cells autophagy by LC3-II accumulation [8]. We questioned whether tamoxifen also triggered autophagy in TNBC cells. Our founding demonstrated that tamoxifen induced endoplasmic reticulum stress (Fig. 3a and b), and stimulation of AMPK/mTOR (Fig. 3c) signaling to trigger autophagy (Fig. 1 and Fig. 2) but had non-significant effect on cell growth inhibition (Fig. 4).

Next, we investigated the role of autophagy induced by tamoxifen or CSC-3436 acted as a protective or pro-death role. Inhibition of autophagy by using shRNA against ATG-5, further increased cell growth inhibition, supporting both tamoxifen and CSC-3436-induced autophagy played a protective role (Fig. 5a). ATG-5 is required for the formation of autophagy and could be truncated to fragment with a molecular mass of 24 kDa mediated by calpain [44, 45]. The cleavage fragment activates caspase-dependent cell death that has been considered to as a contributor to the switch between autophagy and apoptosis. Melanoma differentiation-associated gene 7 (mda-7)/interleukin-24 (IL-24) produced the cleavage of ATG-5 by increasing calpain activity and resulted in autophagy switch to apoptosis [23]. Similarly, our finding demonstrated that CSC-3436 plus tamoxifen treatment produced the cleavage of ATG5 (Fig. 5b).

Undendoplasmic reticulum stressful situations, autophagy could be induced by multiple signaling pathways that related with cell growth, proliferation, survival and death [11, 14]. The serine/threonine kinase mTOR regulates cell survival and proliferation and also known as a major negative regulator of autophagy. Upstream of mTOR, AMPK is a metabolic sensor and activated by low energy. It can inhibit mTOR and lead to autophagy induction [46, 47]. Our finding demonstrated that CSC-3436 decreased slightly tamoxifen-induced up-regulation of p-AMPK protein expression. It may be another mechanism to enhance apoptosis (Fig. 5c).

## Conclusions

Our founding demonstrated two ways which caused CSC-3436 sensitized TNBC MDA-MB-231 breast cancer cells to tamoxifen. CSC-3436 inhibited the effect of tamoxifen on p-AMPK and p-mTOR expressions and caused ATG5-cleavaged which may contribute to autophagy switch to apoptosis. As such, CSC-3436 combined with tamoxifen may be a potential approach for treatment ERα-negative breast cancer, and we suggest that this new strategy be extensively explored.

## Abbreviations

3-MA, 3-methyladenine; AO, acridine orange; ATG, autophagy-related gene; DMEM, Dulbecco’s Modified Eagle Medium; ER, estrogen receptor; FBS, Fetal bovine serine; MTT, 3-(4,5-dimethylthiazol-2-yl)-2,5-diphenyl tetrazolium bromide; PI, propidium iodide; SDS-PAGE, sodium dodecyl sulfate-polyacrylamide gel electrophoresis; SERMs, Selective estrogen receptor modulators; TNBC, Triple-negative breast cancer
